# Crystal structure of chlorido­{tris­[2-(iso­propyl­sulfan­yl)phen­yl]phosphane-κ^4^ *P*,*S*,*S*′,*S*′′}nickel(II) tri­fluoro­methane­sulfonate

**DOI:** 10.1107/S2056989019002068

**Published:** 2019-02-12

**Authors:** Nobuhiro Takeda, Rin Oma, Masafumi Unno

**Affiliations:** a Graduate School of Science and Technology, Gunma University, 1-5-1 Tenjin-cho, Kiryu, Gunma 376-8515, Japan

**Keywords:** crystal structure, nickel, five-coordinate, tripodal tetra­dentate ligand, phosphine, thio­ether

## Abstract

The complex cation of the title compound, [NiCl{P(C_6_H_4_-2-S-*i*-Pr)_3_}](CF_3_SO_3_), has a slightly distorted trigonal–bipyramidal coordination geometry in which three S atoms are located in the equatorial plane, and one P and one Cl atom in the apical positions. In the cation, there are two intra­molecular C—H⋯S hydrogen bonds. In the crystal, there are some inter­molecular C—H⋯O and C—H⋯F hydrogen bonds formed between the cation and the anion. The tri­fluoro­methane­sulfonate anion and one of the methyl groups are both disordered over two sets of sites with occupancies of 0.629 (17):0.371 (17) and 0.786 (14):0.214 (14), respectively.

## Abstract 2

The complex cation of the title compound has a five-coordinate slightly distorted trigonal–bipyramidal structure in which three S atoms are located in the equatorial positions, and one P and one Cl atom in the apical positions.

## Chemical context   

**Figure 1:**
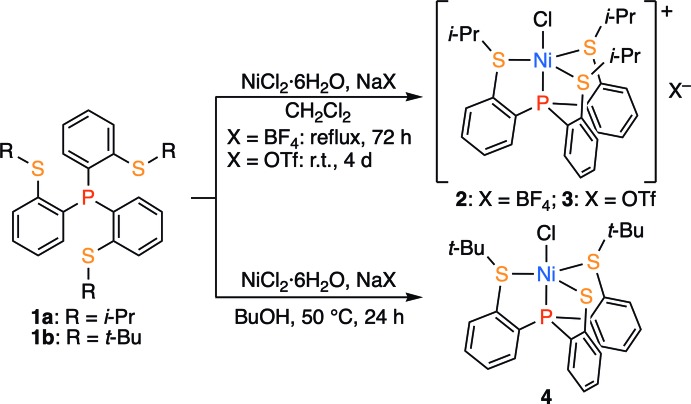
Synthesis of nickel(II) complexes bearing the PS_3_-type tripodal tetra­dentate ligand.

Unusual five-coordinate nickel(II) complexes have been often obtained by use of polydentate ligands such as tripodal tetra­dentate ligands (Orioli, 1971 ▸; Morassi *et al.*, 1973 ▸; Hierso *et al.*, 2003 ▸). A variety of tripodal tetra­dentate ligands having phosphines and/or amines as coordinating sites have been used for the synthesis of five-coordinate nickel(II) complexes. However, for PS_3_-type tripodal tetra­dentate ligands in which three thio­ether moieties are tethered to a phosphine moiety, only one crystal structure (Haugen & Eisenberg, 1969 ▸) had been reported before we started our studies. Recently, we have synthesized new PS_3_-type tripodal tetra­dentate ligands, tris(2-iso­propyl­thio­phen­yl)phosphine, **1a** and tris(2-*tert*-butyl­thio­phen­yl)phosphine, **1b** (Fig. 1 ▸), and reported the syntheses and properties of their group 10 metal complexes (Takeda *et al.*, 2010 ▸, 2016 ▸). Reaction of **1a** with NiCl_2_·6H_2_O in the presence of NaBF_4_ gave the corresponding cationic five-coordinate nickel(II) complex, **2**, while the reaction of **1b** with NiCl_2_·6H_2_O resulted in the elimination of *t*-BuCl to afford a neutral five-coordinate nickel(II) complex, **4** (Fig. 1 ▸). In this paper, we describe the structure of the title compound, [NiCl(*L*)]CF_3_SO_3_ (*L* = **1a**), **3**, which was prepared by reaction of **1a** with NiCl_2_·6H_2_O in the presence of an excess amount of NaCF_3_SO_3_ (Fig. 1 ▸).
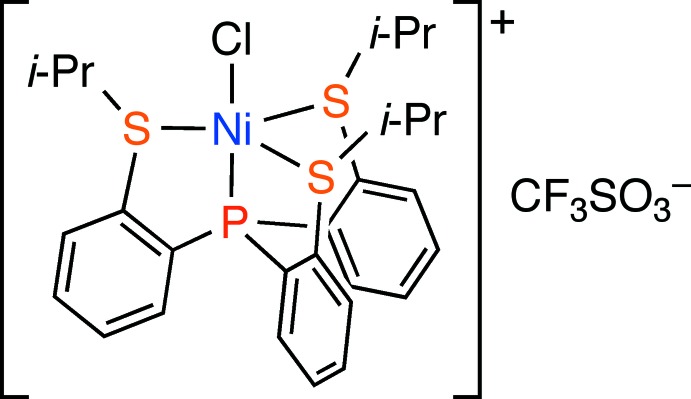


## Structural commentary   

**Figure 2:**
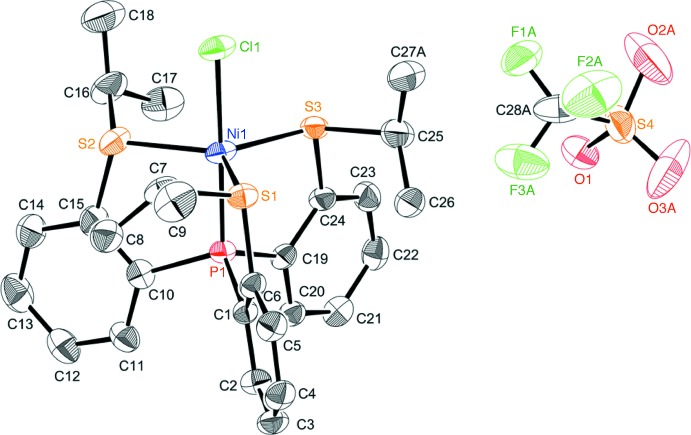
The mol­ecular structure of the title compound, **3**. Displacement ellipsoids are drawn at the 50% probability level. Hydrogen atoms and minor disorder components are omitted for clarity.

**Figure 3:**
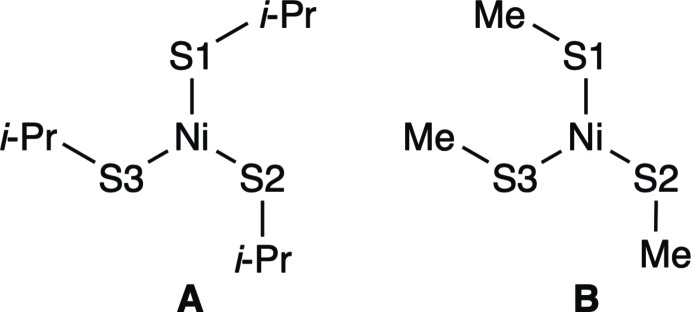
The conformation diagrams of the Ni(S*R*)_3_ moieties (*R* = *i*-Pr or Me) for **3** (**A**) and **5** (**B**), viewed along the Ni—Cl bond.

The structure of the title compound, **3**, is shown in Fig. 2 ▸. The triflate anion and one of the methyl groups are each disordered over two sets of sites with occupancies of 0.629 (17):0.371 (17) and 0.786 (14):0.214 (14), respectively. The complex cation of **3** has a five-coordinate slightly distorted trigonal–bipyramidal structure, in which one P atom and one Cl atom coordinate to the nickel center(II) in the apical positions [P1—Ni1—Cl1 177.83 (5)°] and three S atoms are located in the equatorial positions. In addition, there are two weak C—H⋯S intra­molecular hydrogen bonds (C26—H26*B*⋯S1 and C17—H17*B*⋯S3; Table 1 ▸). Table 2 ▸ presents selected bond lengths and angles of **3** along with those of the related complexes, complex **2** (Takeda *et al.*, 2010 ▸) and the methyl derivative, [NiCl{P(C_6_H_4_-2-SCH_3_)_3_}]ClO_4_, **5** (Haugen & Eisenberg, 1969 ▸). The conformation of the Ni(S-*i*-Pr)_3_ unit of **3** is similar to that of complex **2**, but different from that of **5**, as shown in Fig. 3 ▸. This is probably due to the difference in the bulkiness between the isopropyl and methyl groups. In **3**, the Ni1—S3 bond length [2.3072 (13) Å] is slightly longer than the Ni1—S1 and Ni1—S2 bond lengths [2.2574 (12) and 2.2612 (13) Å, respectively], and the S1—Ni1—S2 bond angle [122.83 (5)°] is slightly larger than the S2—Ni1—S3 and S3—Ni1—S1 bond angles [120.87 (5) and 116.04 (5)°, respectively]. This properties suggests that in complex **3** the five-coordinate trigonal–bipyramidal structure slightly approaches a four-coordinate square-planar structure by the elongation of the Ni—S3 bond. This is a similar tendency to the structure of **2**, and the deviation from trigonal–bipyramidal structure in **3** is smaller than that in **2**. The Ni—S bond lengths of **3** are very close to those of methyl derivative **5**, while the S3—Ni—S1 bond angle of **5** [127.1 (3)°] is large as expected from the conformation **B** (Fig. 3 ▸).

## Supra­molecular features   

**Figure 4:**
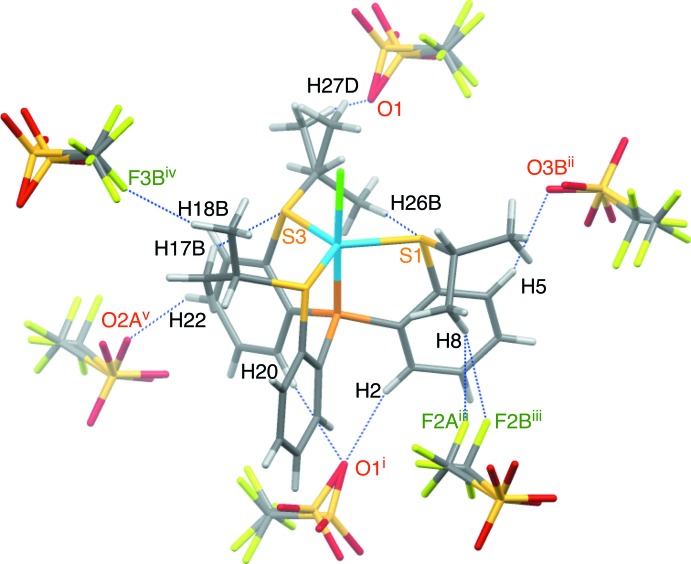
Intra­molecular C—H⋯S hydrogen bonds and inter­molecular C—H⋯O and C—H⋯F hydrogen bonds (blue dashed lines) in **3**. [Symmetry codes: (i) −*x* + 1, −*y* + 1, −*z* + 1; (ii) −*x* + 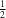, *y* − 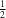, −*z* + 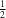; (iii) *x* + 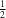, −*y* + 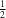, *z* + 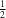; (iv) *x* + 1, *y*, *z*; (v) *x* + 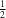, −*y* + 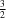, *z* + 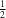.]

**Figure 5:**
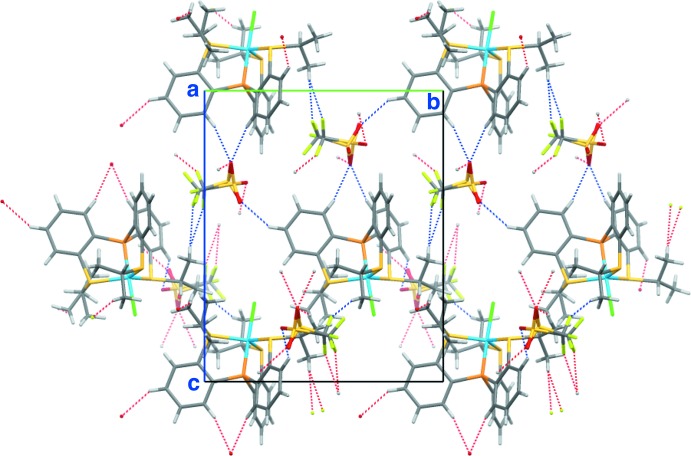
A packing diagram of **3**, viewed along the *a* axis. The C—H⋯O and C—H⋯F hydrogen bonds are shown as dashed lines.

In the crystal of **3**, there are some hydrogen bonds between the cation and the anion (Fig. 4 ▸). The cation and the anion are linked into a tape structure along the *b-*axis direction *via* C—H⋯O and C—H⋯F hydrogen bonds (C2—H2⋯O1^i^, C8—H8⋯F2*A* ^iii^, C8—H8⋯F2*B* ^iii^, C20—H20⋯O1^i^ and C22—H22⋯O2*A* ^v^; symmetry codes as in Table 1 ▸) . The tapes are further linked by weak C—H⋯O and C—H⋯F hydrogen bonds formed between the cation and the minor component of the disordered anion (C5—H5⋯O3*B* ^ii^ and C18—H18*B*⋯F3*B* ^iv^; Table 1 ▸), forming a three-dimensional network (Figs. 5 ▸ and 6 ▸).

## Database survey   

A search of the Cambridge Structural Database (CSD; Groom *et al.*, 2016 ▸) using WebCSD found four structures of nickel complexes having three Ni—S, one Ni—P and one Ni—Cl bonds. The structures of the complexes, [NiCl{P(C_6_H_4_-2-SMe)_3_}]ClO_4_, **5** (refcode: CMTPPN; Haugen & Eisenberg, 1969 ▸) and [NiCl{P(C_6_H_4_-2-S-*i*-Pr)_3_}]BF_4_, **2** (FULMOP; Takeda *et al.*, 2010 ▸), are similar to that of the cationic choloridonickel(II) complex **3**. The structures of the other two complexes, [Ph_3_P=N=PPh_3_][NiCl{P(C_6_H_3_-3-SiMe_3_-2-S)_3_}] (YETYOM; Lee *et al.*, 2006 ▸) and [NiCl{P(C_6_H_4_-2-S)(C_6_H_4_-2-S-*t*-Bu)_2_}], **4** (EZOQAN; Takeda *et al.*, 2016 ▸), are different from that of complex **3**. The former is an anionic nickel(III) complex having three thiol­ato (^−^ *SR*), one chlorido and one phosphine ligands, and the latter, **4**, is a neutral nickel(II) complex having two thio­ether, one thiol­ato, one chlorido and one phosphine ligands.

## Synthesis and crystallization   

A mixture of tris­(2-iso­propyl­thio­phen­yl)phosphine, **1a** (0.141 g, 0.291 mmol), NiCl_2_·6H_2_O (0.060 g, 0.25 mmol) and NaCF_3_SO_3_ (0.345 g, 2.01 mmol) in di­chloro­methane (5 ml) was stirred at room temperature for 4 d. After removal of the solvent under reduced pressure, recrystallization of the residue from a chloro­form/hexane solution gave the title compound, **3**, as blue crystals (0.168 g, 91%).

M.p. 485 K (decomp.) ^1^H NMR (300 MHz, CDCl_3_): *δ* 1.31 (*d*, ^3^ *J*_HH_ = 6.7 Hz, 18H), 3.73 (*sepd*, ^3^ *J*_HH_ = 6.7 Hz, ^4^ *J*_HP_ = 1.6 Hz, 3H), 7.71 (*tdd*, ^3^ *J*_HH_ = 8.3 Hz, *J*_HP_ = 2.2 Hz, ^4^ *J*_HH_ = 1.0 Hz, 3H), 7.80 (*ddd*, ^3^ *J*_HH_ = 8.3 Hz, ^4^ *J*_HP_ = 3.3 Hz, ^4^ *J*_HH_ = 1.0 Hz, 3H), 7.91 (*tdd*, ^3^ *J*_HH_ = 8.3 Hz, *J*_HP_ = 2.5 Hz, ^4^ *J*_HH_ = 1.0 Hz, 3H), 8.68 (*dd*, ^3^ *J*_HH_ = 8.3 Hz, ^3^ *J*_HP_ = 8.3 Hz, 3H). ^13^C{^1^H} NMR (150 MHz, CDCl_3_): *δ* 22.3 (*s*, CH_3_), 50.7 (*s*, CH), 132.9 (*d*, *J*_CP_ = 7.2 Hz, CH), 133.4 (*d*, *J*_CP_ = 13.0 Hz, CH), 133.6 (*s*, CH), 134.8 (*s*, CH), 135.3 (*d*, ^1^ *J*_CP_ =63.6 Hz, C), 137.0 (*d*, ^2^ *J*_CP_ = 23.1 Hz, C), the peak of CF_3_ could not be detected. ^31^P NMR (162 MHz, CDCl_3_): *δ* 103.3. ^19^F NMR (376 MHz, CDCl_3_): *δ* −77.92. IR (KBr): 516.9, 532.3, 551.6, 572.8, 638.4, 673.1, 727.1, 740.6, 779.2, 879.5, 931.6, 1029.9, 1056.9 (S=O), 1116.7, 1157.2 (S=O),1224.7, 1242.1, 1249.8, 1265.2, 1274.9, 1282.6, 1369.4, 1388.7, 1433.0, 1460.0, 1568.0, 1635.5, 2868.0, 2927.7, 2968.2, 3055.0, 3082.0, 3301.9, 3319.3, 3392.6, 3406.1, 3423.4, 3444.6, 3477.4, 3489.0. UV–vis (CHCl_3_): *λ*_max_ 246 (*∊* 35000), 332 (*∊* 5700), 474 (*∊* 350), 639 nm (*∊* 2000). Analysis calculated for C_28_H_33_ClF_3_NiO_3_PS_4_: C 46.20, H 4.57%. Found: C 45.38, H 4.55%.

## Refinement   

Crystal data, data collection and structure refinement details are summarized in Table 3 ▸. The H atoms were positioned geometrically (C—H = 0.95–1.00 Å) and refined as riding atoms with *U*_iso_(H) =1.5*U*_eq_(C) for methyl or 1.2*U*_eq_(C) for aromatic and methine H atoms. The methyl groups were allowed to rotate freely around the C—C bond. The triflate anion exhibits disorder and was modelled with occupancies of 0.629 (17) and 0.371 (17). The geometric parameters of the minor component were restrained to be similar to those of the major component by using *SAME* restraint. In addition, one of the methyl groups in the complex cation exhibits disorder and was modelled with occupancies of 0.786 (14) and 0.214 (14). The C25—C27*A* and C25—C27*B* bond lengths were restrained to be equal to each other by using *SADI* restraint.

## Supplementary Material

Crystal structure: contains datablock(s) I. DOI: 10.1107/S2056989019002068/is5507sup1.cif

Structure factors: contains datablock(s) I. DOI: 10.1107/S2056989019002068/is5507Isup2.hkl

CCDC reference: 1895701

Additional supporting information:  crystallographic information; 3D view; checkCIF report

## Figures and Tables

**Figure 6:**
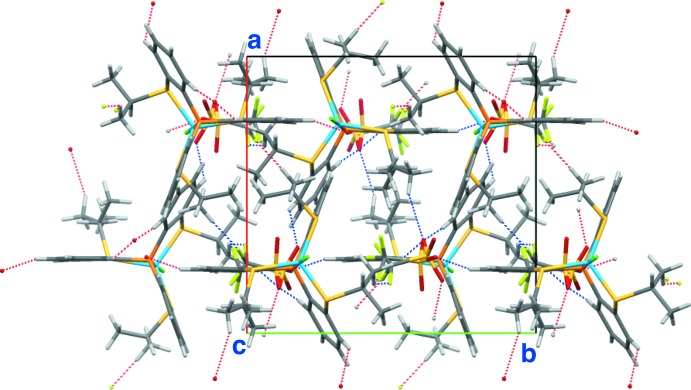
A packing diagram of **3**, viewed along the *c* axis. The C—H⋯O and C—H⋯F hydrogen bonds are shown as dashed lines.

**Table 1:** Hydrogen-bond geometry (Å, °)

*D*—H⋯*A*	*D*—H	H⋯*A*	*D*⋯*A*	*D*—H⋯*A*
C2—H2⋯O1^i^	0.95	2.42	3.336 (5)	162
C5—H5⋯O3*B* ^ii^	0.95	2.55	3.328 (11)	140
C8—H8⋯F2*A* ^iii^	0.98	2.53	3.318 (14)	138
C8—H8⋯F2*B* ^iii^	0.98	2.50	3.30 (2)	139
C17—H17*B*⋯S3	0.98	2.82	3.652 (5)	143
C18—H18*B*⋯F3*B* ^iv^	0.98	2.15	3.099 (12)	162
C20—H20⋯O1^i^	0.95	2.58	3.477 (5)	157
C22—H22⋯O2*A* ^v^	0.95	2.31	3.127 (12)	144
C26—H26*B*⋯S1	0.98	2.85	3.762 (6)	156
C27*B*—H27*D*⋯O1	0.98	2.46	3.308 (19)	144

Symmetry codes: (i) 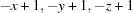; (ii) 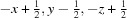; (iii) 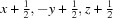; (iv) 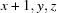; (v) 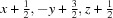.

**Table 2:** Selected bond distances (Å) and angles (°) in complexes **2**, **3** and **5**

compounds	**3**	**2** *^*a*^*	**5** *^*b*^*
Ni1—P1	2.1124 (11)	2.1108 (7)	2.113 (7)
Ni1—S1	2.2574 (12)	2.2454 (7)	2.242 (8)
Ni1—S2	2.2612 (13)	2.2678 (7)	2.269 (6)
Ni1—S3	2.3072 (13)	2.3510 (7)	2.290 (7)
Ni1—Cl	2.2412 (11)	2.2437 (7)	2.227 (7)
			
P1—Ni1—Cl1	177.83 (5)	178.60 (3)	178.5 (3)
S2—Ni1—S3	120.87 (5)	109.53 (3)	112.1 (3)
S3—Ni1—S1	116.04 (5)	119.03 (3)	127.1 (3)
S1—Ni1—S2	122.83 (5)	130.74 (3)	120.6 (2)
P1—Ni1—S	88.16 (4)–88.1 (4)	86.9 – 87.5	88.0 – 88.7
Cl1—Ni1—S	89.88 (4)–93.48 (5)	92.0 – 94.5	90.7 – 92.4

Notes: (*a*) Takeda *et al.* (2010 ▸); (*b*) Haugen & Eisenberg (1969 ▸).

**Table 3:** Experimental details

Crystal data	
Chemical formula	[NiCl(C_27_H_33_PS_3_)](CF_3_SO_3_)
*M*_r_	727.91
Crystal system, space group	Monoclinic, *P*2_1_/*n*
Temperature (K)	120
*a*, *b*, *c* (Å)	13.428 (3), 14.008 (3), 17.110 (3)
β (°)	93.164 (4)
*V* (Å^3^)	3213.5 (11)
*Z*	4
Radiation type	Mo *K*α
μ (mm^−1^)	1.04
Crystal size (mm)	0.10 × 0.08 × 0.02

Data collection	
Diffractometer	Rigaku CrystalClear-SM Expert 2.1 b29
Absorption correction	Numerical (*CrystalClear*; Rigaku, 2013 ▸)
*T*_min_, *T*_max_	0.924, 0.971
No. of measured, independent and observed [*I* > 2σ(*I*)] reflections	51723, 7359, 5054
*R*_int_	0.096
(sin θ/λ)_max_ (Å^−1^)	0.649

Refinement	
*R*[*F* ^2^ > 2σ(*F* ^2^)], *wR*(*F* ^2^), *S*	0.057, 0.144, 1.08
No. of reflections	7359
No. of parameters	451
No. of restraints	20
H-atom treatment	H-atom parameters constrained
Δρ_max_, Δρ_min_ (e Å^−3^)	0.75, −0.35

Computer programs: *CrystalClear* (Rigaku, 2013 ▸), *SHELXT* (Sheldrick, 2015*a* ▸), *SHELXL2014* (Sheldrick, 2015*b* ▸), *Yadokari-XG* (Wakita, 2001 ▸; Kabuto *et al.*, 2009 ▸) and *publCIF* (Westrip, 2010 ▸).

## supplementary crystallographic information

### Crystal data

**Table:**

[NiCl(C_27_H_33_PS_3_)](CF_3_SO_3_)	*F*(000) = 1504
*M**_r_* = 727.91	*D*_x_ = 1.505 Mg m^−^^3^
Monoclinic, *P*2_1_/*n*	Mo *K*α radiation, λ = 0.71075 Å
*a* = 13.428 (3) Å	Cell parameters from 5728 reflections
*b* = 14.008 (3) Å	θ = 2.8–27.5°
*c* = 17.110 (3) Å	µ = 1.04 mm^−^^1^
β = 93.164 (4)°	*T* = 120 K
*V* = 3213.5 (11) Å^3^	Prism, blue
*Z* = 4	0.10 × 0.08 × 0.02 mm

### Data collection

**Table:**

Rigaku CrystalClear-SM Expert 2.1 b29 diffractometer	7359 independent reflections
Radiation source: Rotating Anode	5054 reflections with *I* > 2σ(*I*)
Confocal monochromator	*R*_int_ = 0.096
Detector resolution: 5.8140 pixels mm^-1^	θ_max_ = 27.5°, θ_min_ = 2.8°
profile data from ω–scans	*h* = −17→17
Absorption correction: numerical (CrystalClear; Rigaku, 2013)	*k* = −18→18
*T*_min_ = 0.924, *T*_max_ = 0.971	*l* = −22→22
51723 measured reflections	

### Refinement

**Table:**

Refinement on *F*^2^	20 restraints
Least-squares matrix: full	Hydrogen site location: inferred from neighbouring sites
*R*[*F*^2^ > 2σ(*F*^2^)] = 0.057	H-atom parameters constrained
*wR*(*F*^2^) = 0.144	*w* = 1/[σ^2^(*F*_o_^2^) + (0.0517*P*)^2^ + 5.7574*P*] where *P* = (*F*_o_^2^ + 2*F*_c_^2^)/3
*S* = 1.08	(Δ/σ)_max_ < 0.001
7359 reflections	Δρ_max_ = 0.75 e Å^−^^3^
451 parameters	Δρ_min_ = −0.35 e Å^−^^3^

### Special details

**Table:**

Geometry. All esds (except the esd in the dihedral angle between two l.s. planes) are estimated using the full covariance matrix. The cell esds are taken into account individually in the estimation of esds in distances, angles and torsion angles; correlations between esds in cell parameters are only used when they are defined by crystal symmetry. An approximate (isotropic) treatment of cell esds is used for estimating esds involving l.s. planes.

### Fractional atomic coordinates and isotropic or equivalent isotropic displacement parameters (Å^2^)

**Table:**

	*x*	*y*	*z*	*U*_iso_*/*U*_eq_	Occ. (<1)
Ni1	0.75663 (4)	0.31726 (4)	0.35830 (3)	0.02972 (15)	
Cl1	0.77545 (8)	0.29069 (9)	0.23080 (6)	0.0409 (3)	
P1	0.73682 (7)	0.33699 (7)	0.47886 (5)	0.0246 (2)	
C1	0.6229 (3)	0.2789 (3)	0.5066 (2)	0.0262 (8)	
C2	0.5845 (3)	0.2845 (3)	0.5811 (2)	0.0321 (9)	
H2	0.6179	0.3205	0.6216	0.039*	
C3	0.4967 (3)	0.2363 (3)	0.5944 (3)	0.0384 (10)	
H3	0.4706	0.2389	0.6448	0.046*	
C4	0.4466 (3)	0.1846 (3)	0.5360 (3)	0.0381 (10)	
H4	0.3872	0.1516	0.5468	0.046*	
C5	0.4822 (3)	0.1805 (3)	0.4619 (3)	0.0358 (10)	
H5	0.4465	0.1470	0.4210	0.043*	
C6	0.5721 (3)	0.2269 (3)	0.4482 (2)	0.0274 (8)	
S1	0.61937 (8)	0.22394 (8)	0.35266 (6)	0.0304 (2)	
C7	0.6643 (3)	0.0991 (3)	0.3441 (3)	0.0386 (10)	
H7	0.7114	0.0985	0.3008	0.046*	
C8	0.7218 (4)	0.0635 (4)	0.4164 (3)	0.0491 (12)	
H8	0.6777	0.0613	0.4601	0.074*	
H8A	0.7778	0.1066	0.4294	0.074*	
H8B	0.7473	−0.0008	0.4067	0.074*	
C9	0.5777 (4)	0.0336 (4)	0.3189 (3)	0.0570 (14)	
H9	0.6037	−0.0287	0.3038	0.085*	
H9A	0.5396	0.0620	0.2743	0.085*	
H9B	0.5342	0.0254	0.3625	0.085*	
C10	0.8429 (3)	0.2904 (3)	0.5377 (2)	0.0297 (9)	
C11	0.8506 (4)	0.2885 (3)	0.6188 (2)	0.0364 (10)	
H11	0.7951	0.3060	0.6479	0.044*	
C12	0.9387 (4)	0.2611 (4)	0.6570 (3)	0.0460 (12)	
H12	0.9448	0.2618	0.7126	0.055*	
C13	1.0190 (4)	0.2325 (4)	0.6148 (3)	0.0515 (13)	
H13	1.0798	0.2145	0.6418	0.062*	
C14	1.0113 (3)	0.2299 (4)	0.5342 (3)	0.0471 (12)	
H14	1.0653	0.2075	0.5056	0.057*	
C15	0.9229 (3)	0.2607 (3)	0.4950 (3)	0.0364 (10)	
S2	0.91100 (8)	0.26050 (9)	0.39095 (6)	0.0383 (3)	
C16	1.0134 (4)	0.3448 (4)	0.3714 (3)	0.0515 (13)	
H16	1.0729	0.3224	0.4041	0.062*	
C17	0.9930 (4)	0.4436 (4)	0.3978 (3)	0.0529 (13)	
H17	1.0532	0.4825	0.3946	0.079*	
H17A	0.9732	0.4421	0.4521	0.079*	
H17B	0.9390	0.4713	0.3643	0.079*	
C18	1.0401 (4)	0.3352 (5)	0.2900 (3)	0.0659 (16)	
H18	0.9819	0.3502	0.2550	0.099*	
H18A	1.0617	0.2696	0.2805	0.099*	
H18B	1.0945	0.3794	0.2799	0.099*	
C19	0.7307 (3)	0.4636 (3)	0.5007 (2)	0.0270 (8)	
C20	0.7291 (3)	0.5015 (3)	0.5767 (2)	0.0297 (9)	
H20	0.7287	0.4601	0.6206	0.036*	
C21	0.7283 (3)	0.5992 (3)	0.5871 (3)	0.0341 (9)	
H21	0.7258	0.6248	0.6383	0.041*	
C22	0.7312 (3)	0.6603 (3)	0.5236 (3)	0.0370 (10)	
H22	0.7312	0.7274	0.5315	0.044*	
C23	0.7340 (3)	0.6238 (3)	0.4487 (3)	0.0372 (10)	
H23	0.7375	0.6657	0.4053	0.045*	
C24	0.7317 (3)	0.5263 (3)	0.4371 (2)	0.0288 (9)	
S3	0.72947 (8)	0.47865 (8)	0.34063 (6)	0.0310 (2)	
C25	0.6003 (3)	0.5122 (5)	0.3077 (3)	0.0551 (15)	
H25A	0.5974	0.5834	0.3110	0.066*	0.786 (14)
H25B	0.5936	0.4535	0.2745	0.066*	0.214 (14)
C26	0.5214 (3)	0.4751 (4)	0.3611 (3)	0.0459 (12)	
H26	0.4550	0.4935	0.3396	0.069*	
H26A	0.5329	0.5027	0.4135	0.069*	
H26B	0.5257	0.4054	0.3645	0.069*	
C27A	0.5825 (5)	0.4890 (6)	0.2262 (3)	0.049 (2)	0.786 (14)
H27A	0.5843	0.4195	0.2195	0.074*	0.786 (14)
H27B	0.6342	0.5184	0.1959	0.074*	0.786 (14)
H27C	0.5170	0.5132	0.2076	0.074*	0.786 (14)
C27B	0.5769 (15)	0.5677 (16)	0.2455 (12)	0.040 (7)	0.214 (14)
H27D	0.5064	0.5589	0.2292	0.059*	0.214 (14)
H27E	0.6184	0.5502	0.2024	0.059*	0.214 (14)
H27F	0.5887	0.6348	0.2596	0.059*	0.214 (14)
S4A	0.2594 (6)	0.5996 (6)	0.1890 (4)	0.0617 (17)	0.629 (17)
O1	0.3353 (3)	0.6108 (2)	0.25204 (19)	0.0508 (9)	
O2A	0.2747 (16)	0.6526 (8)	0.1192 (6)	0.119 (6)	0.629 (17)
O3A	0.1589 (6)	0.6058 (12)	0.2106 (7)	0.114 (6)	0.629 (17)
C28A	0.2734 (13)	0.4774 (10)	0.1571 (6)	0.061 (6)	0.629 (17)
F1A	0.3485 (7)	0.4632 (8)	0.1181 (5)	0.070 (2)	0.629 (17)
F2A	0.1947 (10)	0.4527 (12)	0.1075 (7)	0.104 (5)	0.629 (17)
F3A	0.2727 (9)	0.4177 (5)	0.2155 (4)	0.083 (3)	0.629 (17)
S4B	0.2809 (6)	0.6234 (5)	0.1799 (4)	0.0279 (14)	0.371 (17)
O2B	0.3381 (11)	0.6469 (12)	0.1159 (7)	0.056 (4)	0.371 (17)
O3B	0.1917 (9)	0.6754 (11)	0.1844 (6)	0.055 (4)	0.371 (17)
C28B	0.2460 (16)	0.4994 (15)	0.1619 (11)	0.060 (9)	0.371 (17)
F1B	0.320 (2)	0.4458 (15)	0.152 (2)	0.160 (17)	0.371 (17)
F2B	0.1807 (14)	0.4953 (11)	0.0986 (11)	0.061 (4)	0.371 (17)
F3B	0.1961 (17)	0.4693 (15)	0.2206 (8)	0.116 (10)	0.371 (17)

### Atomic displacement parameters (Å^2^)

**Table:**

	*U*^11^	*U*^22^	*U*^33^	*U*^12^	*U*^13^	*U*^23^
Ni1	0.0264 (3)	0.0409 (3)	0.0222 (3)	−0.0057 (2)	0.0052 (2)	−0.0053 (2)
Cl1	0.0365 (6)	0.0615 (8)	0.0254 (5)	−0.0050 (5)	0.0077 (4)	−0.0094 (5)
P1	0.0243 (5)	0.0283 (5)	0.0214 (5)	−0.0028 (4)	0.0023 (4)	−0.0017 (4)
C1	0.024 (2)	0.027 (2)	0.028 (2)	0.0007 (16)	0.0046 (15)	0.0029 (16)
C2	0.035 (2)	0.033 (2)	0.029 (2)	−0.0028 (19)	0.0064 (17)	−0.0028 (17)
C3	0.044 (3)	0.035 (2)	0.038 (2)	0.001 (2)	0.019 (2)	0.0055 (19)
C4	0.030 (2)	0.034 (2)	0.051 (3)	−0.0036 (19)	0.014 (2)	0.006 (2)
C5	0.031 (2)	0.034 (2)	0.042 (2)	−0.0056 (19)	0.0038 (18)	−0.0005 (19)
C6	0.022 (2)	0.029 (2)	0.032 (2)	−0.0018 (17)	0.0046 (16)	0.0003 (16)
S1	0.0306 (5)	0.0352 (6)	0.0255 (5)	−0.0062 (4)	0.0021 (4)	−0.0049 (4)
C7	0.045 (3)	0.035 (2)	0.038 (2)	−0.005 (2)	0.014 (2)	−0.0136 (19)
C8	0.056 (3)	0.042 (3)	0.051 (3)	0.008 (2)	0.014 (2)	0.002 (2)
C9	0.065 (4)	0.038 (3)	0.068 (4)	−0.014 (3)	0.009 (3)	−0.020 (3)
C10	0.028 (2)	0.027 (2)	0.034 (2)	−0.0057 (17)	−0.0026 (17)	−0.0012 (16)
C11	0.047 (3)	0.032 (2)	0.030 (2)	0.001 (2)	−0.0038 (19)	0.0004 (17)
C12	0.052 (3)	0.046 (3)	0.039 (3)	0.001 (2)	−0.010 (2)	0.005 (2)
C13	0.039 (3)	0.059 (3)	0.054 (3)	−0.002 (3)	−0.015 (2)	0.009 (3)
C14	0.027 (2)	0.063 (3)	0.052 (3)	0.005 (2)	0.001 (2)	0.008 (2)
C15	0.027 (2)	0.041 (3)	0.041 (2)	−0.0025 (19)	−0.0005 (18)	0.0003 (19)
S2	0.0317 (6)	0.0464 (7)	0.0379 (6)	0.0025 (5)	0.0105 (4)	−0.0053 (5)
C16	0.028 (2)	0.068 (4)	0.061 (3)	−0.005 (2)	0.018 (2)	0.002 (3)
C17	0.040 (3)	0.064 (4)	0.055 (3)	−0.022 (3)	0.007 (2)	−0.005 (3)
C18	0.057 (4)	0.091 (5)	0.052 (3)	−0.002 (3)	0.020 (3)	−0.004 (3)
C19	0.0209 (19)	0.030 (2)	0.031 (2)	−0.0013 (16)	0.0051 (15)	−0.0012 (16)
C20	0.025 (2)	0.035 (2)	0.029 (2)	−0.0009 (17)	0.0050 (16)	0.0011 (16)
C21	0.032 (2)	0.033 (2)	0.038 (2)	−0.0042 (19)	0.0028 (18)	−0.0078 (18)
C22	0.031 (2)	0.029 (2)	0.051 (3)	0.0009 (18)	0.0029 (19)	−0.0011 (19)
C23	0.036 (2)	0.031 (2)	0.044 (3)	0.0018 (19)	0.0061 (19)	0.0096 (19)
C24	0.025 (2)	0.035 (2)	0.027 (2)	−0.0007 (17)	0.0060 (16)	0.0035 (16)
S3	0.0256 (5)	0.0430 (6)	0.0247 (5)	−0.0003 (4)	0.0048 (4)	0.0063 (4)
C25	0.029 (3)	0.096 (4)	0.040 (3)	0.009 (3)	0.002 (2)	0.022 (3)
C26	0.024 (2)	0.067 (3)	0.046 (3)	0.005 (2)	0.0067 (19)	0.014 (2)
C27A	0.034 (3)	0.079 (6)	0.035 (3)	−0.003 (3)	0.000 (3)	−0.005 (3)
C27B	0.022 (11)	0.032 (14)	0.065 (16)	0.010 (9)	0.010 (10)	0.005 (11)
S4A	0.082 (4)	0.063 (3)	0.0385 (19)	0.032 (3)	−0.009 (2)	−0.001 (2)
O1	0.059 (2)	0.054 (2)	0.0383 (18)	−0.0034 (18)	−0.0051 (16)	0.0042 (15)
O2A	0.229 (17)	0.066 (6)	0.057 (6)	0.046 (10)	−0.024 (8)	0.009 (4)
O3A	0.059 (5)	0.171 (15)	0.111 (8)	0.051 (7)	−0.012 (5)	−0.075 (9)
C28A	0.078 (12)	0.074 (12)	0.033 (7)	−0.028 (10)	0.015 (7)	−0.016 (8)
F1A	0.079 (5)	0.070 (5)	0.065 (4)	0.014 (3)	0.029 (4)	−0.008 (3)
F2A	0.093 (7)	0.151 (12)	0.066 (6)	−0.020 (8)	−0.005 (5)	−0.050 (8)
F3A	0.135 (8)	0.055 (4)	0.061 (4)	−0.023 (5)	0.026 (4)	0.002 (3)
S4B	0.036 (2)	0.032 (3)	0.015 (2)	−0.0002 (19)	0.0013 (14)	0.0037 (16)
O2B	0.069 (9)	0.068 (9)	0.031 (6)	−0.027 (8)	0.007 (6)	0.018 (5)
O3B	0.051 (7)	0.065 (9)	0.046 (6)	0.024 (6)	−0.013 (5)	−0.013 (6)
C28B	0.048 (12)	0.060 (13)	0.070 (19)	0.008 (10)	−0.023 (11)	0.021 (11)
F1B	0.25 (4)	0.056 (12)	0.17 (3)	0.068 (17)	−0.03 (2)	−0.033 (16)
F2B	0.054 (7)	0.066 (9)	0.061 (7)	−0.004 (6)	−0.017 (5)	−0.025 (6)
F3B	0.153 (18)	0.106 (15)	0.086 (9)	−0.096 (15)	−0.020 (9)	0.047 (8)

### Geometric parameters (Å, º)

**Table:**

Ni1—P1	2.1124 (11)	C17—H17A	0.9800
Ni1—Cl1	2.2412 (11)	C17—H17B	0.9800
Ni1—S1	2.2574 (12)	C18—H18	0.9800
Ni1—S2	2.2612 (13)	C18—H18A	0.9800
Ni1—S3	2.3072 (13)	C18—H18B	0.9800
P1—C19	1.815 (4)	C19—C24	1.398 (5)
P1—C1	1.819 (4)	C19—C20	1.406 (5)
P1—C10	1.820 (4)	C20—C21	1.380 (6)
C1—C6	1.385 (5)	C20—H20	0.9500
C1—C2	1.403 (5)	C21—C22	1.385 (6)
C2—C3	1.388 (6)	C21—H21	0.9500
C2—H2	0.9500	C22—C23	1.382 (6)
C3—C4	1.379 (6)	C22—H22	0.9500
C3—H3	0.9500	C23—C24	1.380 (6)
C4—C5	1.381 (6)	C23—H23	0.9500
C4—H4	0.9500	C24—S3	1.779 (4)
C5—C6	1.401 (6)	S3—C25	1.854 (5)
C5—H5	0.9500	C25—C27B	1.341 (15)
C6—S1	1.787 (4)	C25—C27A	1.440 (7)
S1—C7	1.858 (5)	C25—C26	1.528 (6)
C7—C8	1.507 (7)	C25—H25A	1.0000
C7—C9	1.524 (7)	C25—H25B	1.0000
C7—H7	1.0000	C26—H26	0.9800
C8—H8	0.9800	C26—H26A	0.9800
C8—H8A	0.9800	C26—H26B	0.9800
C8—H8B	0.9800	C27A—H27A	0.9800
C9—H9	0.9800	C27A—H27B	0.9800
C9—H9A	0.9800	C27A—H27C	0.9800
C9—H9B	0.9800	C27B—H27D	0.9800
C10—C11	1.386 (6)	C27B—H27E	0.9800
C10—C15	1.396 (6)	C27B—H27F	0.9800
C11—C12	1.375 (6)	S4A—O3A	1.421 (8)
C11—H11	0.9500	S4A—O2A	1.432 (9)
C12—C13	1.390 (7)	S4A—O1	1.450 (7)
C12—H12	0.9500	S4A—C28A	1.809 (14)
C13—C14	1.378 (7)	O1—S4B	1.410 (8)
C13—H13	0.9500	C28A—F1A	1.255 (19)
C14—C15	1.398 (6)	C28A—F3A	1.302 (13)
C14—H14	0.9500	C28A—F2A	1.364 (14)
C15—S2	1.779 (4)	S4B—O3B	1.408 (11)
S2—C16	1.857 (5)	S4B—O2B	1.410 (10)
C16—C18	1.462 (7)	S4B—C28B	1.822 (17)
C16—C17	1.487 (8)	C28B—F1B	1.26 (2)
C16—H16	1.0000	C28B—F3B	1.307 (17)
C17—H17	0.9800	C28B—F2B	1.357 (17)
			
P1—Ni1—Cl1	177.83 (5)	H17—C17—H17A	109.5
P1—Ni1—S1	88.34 (4)	C16—C17—H17B	109.5
Cl1—Ni1—S1	89.88 (4)	H17—C17—H17B	109.5
P1—Ni1—S2	88.16 (4)	H17A—C17—H17B	109.5
Cl1—Ni1—S2	91.78 (4)	C16—C18—H18	109.5
S1—Ni1—S2	122.83 (5)	C16—C18—H18A	109.5
P1—Ni1—S3	88.41 (4)	H18—C18—H18A	109.5
Cl1—Ni1—S3	93.48 (5)	C16—C18—H18B	109.5
S1—Ni1—S3	116.04 (5)	H18—C18—H18B	109.5
S2—Ni1—S3	120.87 (5)	H18A—C18—H18B	109.5
C19—P1—C1	109.59 (18)	C24—C19—C20	119.0 (4)
C19—P1—C10	106.25 (18)	C24—C19—P1	116.8 (3)
C1—P1—C10	109.81 (18)	C20—C19—P1	124.2 (3)
C19—P1—Ni1	109.76 (13)	C21—C20—C19	119.6 (4)
C1—P1—Ni1	110.45 (13)	C21—C20—H20	120.2
C10—P1—Ni1	110.90 (14)	C19—C20—H20	120.2
C6—C1—C2	119.5 (4)	C20—C21—C22	120.8 (4)
C6—C1—P1	115.8 (3)	C20—C21—H21	119.6
C2—C1—P1	124.7 (3)	C22—C21—H21	119.6
C3—C2—C1	118.7 (4)	C23—C22—C21	120.1 (4)
C3—C2—H2	120.7	C23—C22—H22	120.0
C1—C2—H2	120.7	C21—C22—H22	120.0
C4—C3—C2	121.4 (4)	C24—C23—C22	119.9 (4)
C4—C3—H3	119.3	C24—C23—H23	120.1
C2—C3—H3	119.3	C22—C23—H23	120.1
C3—C4—C5	120.5 (4)	C23—C24—C19	120.7 (4)
C3—C4—H4	119.7	C23—C24—S3	120.2 (3)
C5—C4—H4	119.7	C19—C24—S3	119.1 (3)
C4—C5—C6	118.6 (4)	C24—S3—C25	98.9 (2)
C4—C5—H5	120.7	C24—S3—Ni1	104.55 (14)
C6—C5—H5	120.7	C25—S3—Ni1	115.2 (2)
C1—C6—C5	121.2 (4)	C27B—C25—C26	122.1 (9)
C1—C6—S1	119.1 (3)	C27A—C25—C26	114.8 (5)
C5—C6—S1	119.7 (3)	C27B—C25—S3	124.2 (9)
C6—S1—C7	103.16 (19)	C27A—C25—S3	109.8 (4)
C6—S1—Ni1	106.14 (13)	C26—C25—S3	113.6 (3)
C7—S1—Ni1	106.29 (15)	C27A—C25—H25A	105.9
C8—C7—C9	112.4 (4)	C26—C25—H25A	105.9
C8—C7—S1	113.6 (3)	S3—C25—H25A	105.9
C9—C7—S1	110.1 (3)	C27B—C25—H25B	91.0
C8—C7—H7	106.8	C26—C25—H25B	91.0
C9—C7—H7	106.8	S3—C25—H25B	91.0
S1—C7—H7	106.8	C25—C26—H26	109.5
C7—C8—H8	109.5	C25—C26—H26A	109.5
C7—C8—H8A	109.5	H26—C26—H26A	109.5
H8—C8—H8A	109.5	C25—C26—H26B	109.5
C7—C8—H8B	109.5	H26—C26—H26B	109.5
H8—C8—H8B	109.5	H26A—C26—H26B	109.5
H8A—C8—H8B	109.5	C25—C27A—H27A	109.5
C7—C9—H9	109.5	C25—C27A—H27B	109.5
C7—C9—H9A	109.5	H27A—C27A—H27B	109.5
H9—C9—H9A	109.5	C25—C27A—H27C	109.5
C7—C9—H9B	109.5	H27A—C27A—H27C	109.5
H9—C9—H9B	109.5	H27B—C27A—H27C	109.5
H9A—C9—H9B	109.5	C25—C27B—H27D	109.5
C11—C10—C15	120.1 (4)	C25—C27B—H27E	109.5
C11—C10—P1	125.0 (3)	H27D—C27B—H27E	109.5
C15—C10—P1	114.7 (3)	C25—C27B—H27F	109.5
C12—C11—C10	119.7 (4)	H27D—C27B—H27F	109.5
C12—C11—H11	120.1	H27E—C27B—H27F	109.5
C10—C11—H11	120.1	O3A—S4A—O2A	111.6 (8)
C11—C12—C13	120.4 (4)	O3A—S4A—O1	116.0 (5)
C11—C12—H12	119.8	O2A—S4A—O1	115.9 (7)
C13—C12—H12	119.8	O3A—S4A—C28A	104.6 (8)
C14—C13—C12	120.7 (4)	O2A—S4A—C28A	102.6 (6)
C14—C13—H13	119.6	O1—S4A—C28A	104.2 (6)
C12—C13—H13	119.6	F1A—C28A—F3A	110.3 (14)
C13—C14—C15	119.1 (5)	F1A—C28A—F2A	104.3 (11)
C13—C14—H14	120.4	F3A—C28A—F2A	106.0 (10)
C15—C14—H14	120.4	F1A—C28A—S4A	114.2 (9)
C10—C15—C14	119.9 (4)	F3A—C28A—S4A	111.8 (9)
C10—C15—S2	119.7 (3)	F2A—C28A—S4A	109.7 (12)
C14—C15—S2	120.4 (3)	O3B—S4B—O1	114.7 (7)
C15—S2—C16	98.8 (2)	O3B—S4B—O2B	115.0 (9)
C15—S2—Ni1	106.11 (14)	O1—S4B—O2B	115.6 (7)
C16—S2—Ni1	114.20 (18)	O3B—S4B—C28B	106.9 (9)
C18—C16—C17	115.8 (5)	O1—S4B—C28B	98.3 (7)
C18—C16—S2	109.6 (4)	O2B—S4B—C28B	103.7 (9)
C17—C16—S2	112.7 (3)	F1B—C28B—F3B	111 (2)
C18—C16—H16	106.0	F1B—C28B—F2B	110.3 (19)
C17—C16—H16	106.0	F3B—C28B—F2B	105.3 (16)
S2—C16—H16	106.0	F1B—C28B—S4B	113.1 (17)
C16—C17—H17	109.5	F3B—C28B—S4B	108.4 (13)
C16—C17—H17A	109.5	F2B—C28B—S4B	109.0 (14)
			
C19—P1—C1—C6	125.3 (3)	C15—S2—C16—C17	−66.9 (4)
C10—P1—C1—C6	−118.4 (3)	Ni1—S2—C16—C17	45.3 (4)
Ni1—P1—C1—C6	4.2 (3)	C1—P1—C19—C24	−116.7 (3)
C19—P1—C1—C2	−54.1 (4)	C10—P1—C19—C24	124.7 (3)
C10—P1—C1—C2	62.3 (4)	Ni1—P1—C19—C24	4.8 (3)
Ni1—P1—C1—C2	−175.1 (3)	C1—P1—C19—C20	66.1 (4)
C6—C1—C2—C3	0.9 (6)	C10—P1—C19—C20	−52.5 (4)
P1—C1—C2—C3	−179.8 (3)	Ni1—P1—C19—C20	−172.5 (3)
C1—C2—C3—C4	−0.8 (7)	C24—C19—C20—C21	0.2 (6)
C2—C3—C4—C5	−0.8 (7)	P1—C19—C20—C21	177.5 (3)
C3—C4—C5—C6	2.4 (7)	C19—C20—C21—C22	−1.4 (6)
C2—C1—C6—C5	0.7 (6)	C20—C21—C22—C23	0.5 (7)
P1—C1—C6—C5	−178.6 (3)	C21—C22—C23—C24	1.4 (7)
C2—C1—C6—S1	177.8 (3)	C22—C23—C24—C19	−2.6 (6)
P1—C1—C6—S1	−1.6 (4)	C22—C23—C24—S3	177.1 (3)
C4—C5—C6—C1	−2.3 (6)	C20—C19—C24—C23	1.7 (6)
C4—C5—C6—S1	−179.4 (3)	P1—C19—C24—C23	−175.7 (3)
C1—C6—S1—C7	110.1 (3)	C20—C19—C24—S3	−177.9 (3)
C5—C6—S1—C7	−72.9 (4)	P1—C19—C24—S3	4.7 (4)
C1—C6—S1—Ni1	−1.5 (4)	C23—C24—S3—C25	−71.4 (4)
C5—C6—S1—Ni1	175.6 (3)	C19—C24—S3—C25	108.3 (4)
C6—S1—C7—C8	−43.9 (4)	C23—C24—S3—Ni1	169.5 (3)
Ni1—S1—C7—C8	67.5 (3)	C19—C24—S3—Ni1	−10.9 (3)
C6—S1—C7—C9	83.1 (4)	C24—S3—C25—C27B	121.1 (14)
Ni1—S1—C7—C9	−165.4 (3)	Ni1—S3—C25—C27B	−128.1 (14)
C19—P1—C10—C11	63.9 (4)	C24—S3—C25—C27A	174.4 (5)
C1—P1—C10—C11	−54.5 (4)	Ni1—S3—C25—C27A	−74.8 (5)
Ni1—P1—C10—C11	−176.9 (3)	C24—S3—C25—C26	−55.5 (5)
C19—P1—C10—C15	−112.0 (3)	Ni1—S3—C25—C26	55.3 (5)
C1—P1—C10—C15	129.6 (3)	O3A—S4A—C28A—F1A	163.6 (10)
Ni1—P1—C10—C15	7.2 (4)	O2A—S4A—C28A—F1A	47.0 (12)
C15—C10—C11—C12	2.7 (6)	O1—S4A—C28A—F1A	−74.3 (10)
P1—C10—C11—C12	−172.9 (3)	O3A—S4A—C28A—F3A	−70.4 (13)
C10—C11—C12—C13	−2.0 (7)	O2A—S4A—C28A—F3A	173.0 (12)
C11—C12—C13—C14	−0.8 (8)	O1—S4A—C28A—F3A	51.8 (13)
C12—C13—C14—C15	2.9 (8)	O3A—S4A—C28A—F2A	46.9 (11)
C11—C10—C15—C14	−0.6 (7)	O2A—S4A—C28A—F2A	−69.7 (12)
P1—C10—C15—C14	175.5 (4)	O1—S4A—C28A—F2A	169.1 (9)
C11—C10—C15—S2	178.4 (3)	O3B—S4B—C28B—F1B	176 (2)
P1—C10—C15—S2	−5.5 (5)	O1—S4B—C28B—F1B	−65 (2)
C13—C14—C15—C10	−2.2 (7)	O2B—S4B—C28B—F1B	54 (2)
C13—C14—C15—S2	178.8 (4)	O3B—S4B—C28B—F3B	−61.0 (16)
C10—C15—S2—C16	119.9 (4)	O1—S4B—C28B—F3B	58.1 (15)
C14—C15—S2—C16	−61.1 (4)	O2B—S4B—C28B—F3B	177.1 (14)
C10—C15—S2—Ni1	1.5 (4)	O3B—S4B—C28B—F2B	53.1 (16)
C14—C15—S2—Ni1	−179.5 (4)	O1—S4B—C28B—F2B	172.2 (14)
C15—S2—C16—C18	162.6 (4)	O2B—S4B—C28B—F2B	−68.9 (16)
Ni1—S2—C16—C18	−85.2 (4)		

### Hydrogen-bond geometry (Å, º)

**Table:**

*D*—H···*A*	*D*—H	H···*A*	*D*···*A*	*D*—H···*A*
C2—H2···O1^i^	0.95	2.42	3.336 (5)	162
C5—H5···O3*B*^ii^	0.95	2.55	3.328 (11)	140
C8—H8···F2*A*^iii^	0.98	2.53	3.318 (14)	138
C8—H8···F2*B*^iii^	0.98	2.50	3.30 (2)	139
C17—H17*B*···S3	0.98	2.82	3.652 (5)	143
C18—H18*B*···F3*B*^iv^	0.98	2.15	3.099 (12)	162
C20—H20···O1^i^	0.95	2.58	3.477 (5)	157
C22—H22···O2*A*^v^	0.95	2.31	3.127 (12)	144
C26—H26*B*···S1	0.98	2.85	3.762 (6)	156
C27*B*—H27*D*···O1	0.98	2.46	3.308 (19)	144

Symmetry codes: (i) −*x*+1, −*y*+1, −*z*+1; (ii) −*x*+1/2, *y*−1/2, −*z*+1/2; (iii) *x*+1/2, −*y*+1/2, *z*+1/2; (iv) *x*+1, *y*, *z*; (v) *x*+1/2, −*y*+3/2, *z*+1/2.
